# Bringing Induced Pluripotent Stem Cell Technology to the Bedside

**DOI:** 10.31662/jmaj.2018-0005

**Published:** 2018-09-28

**Authors:** Peter Karagiannis, Ayaka Nakauchi, Shinya Yamanaka

**Affiliations:** 1 Center for iPS Cell Research and Application, Kyoto University, Kyoto, Japan

**Keywords:** cell reprogramming, drug discovery, induced pluripotent stem cells, regenerative medicine

## Abstract

Induced pluripotent stem cells (iPSCs) describe somatic cells that have been reprogrammed to the pluripotent state. From a scientific perspective, their discovery has provided a molecular roadmap for turning on and off cell identities, effectively allowing any cell type to have its identity changed into any other cell type. They also act as a human model for understanding the development of every cell and organ in the body. In addition, because they can be prepared from patients, iPSCs offer a unique human model for studying disease development, including many diseases that are generally diagnosed at a late stage of their development. These models have provided new insights on the pathogenesis and new targets to prevent or reverse the disease development process. Indeed, clinical studies on compounds based on drug screening hits in human iPSC disease models have begun. Because of their proliferation and differentiation capacity, iPSCs can also be used to prepare cells for transplantations, and related clinical studies using iPSC-based cell therapies are ongoing. The combination of iPSCs with other technologies or therapeutic strategies is expected to expand their medical benefits. In this review, we consider medical accomplishments based on iPSC research and future ones that can be anticipated.

## Introduction

**Figure 1.**
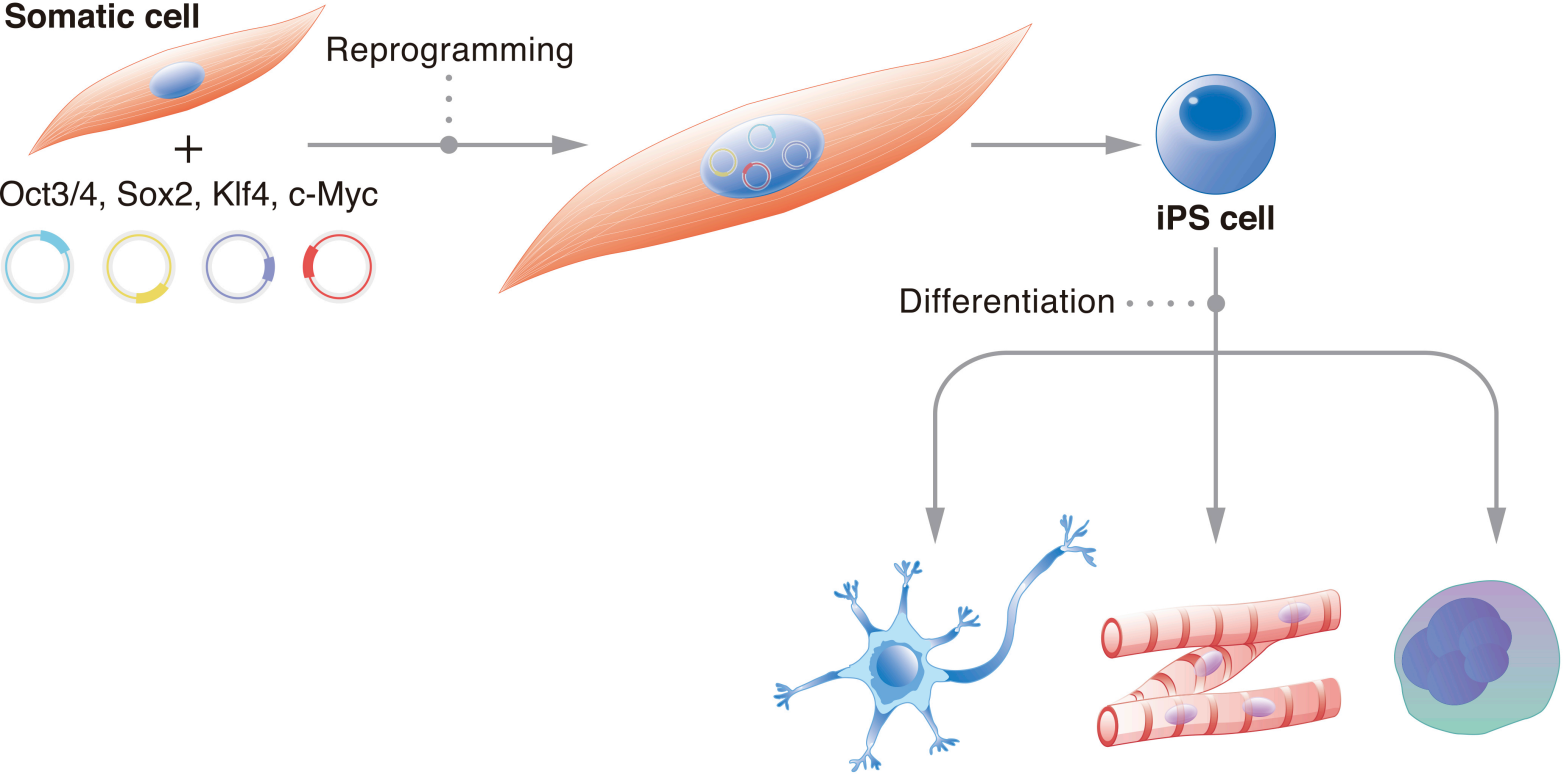
iPSCs describe cells that have been reprogrammed to the pluripotent state. In the illustration, a somatic cell has had OSKM exogenously expressed to initiate the reprogramming mechanism. The result is an iPSC, which in proper culture conditions can be induced to differentiate into any cell type.

It is estimated that the human body consists of trillions of cells. Each one carries a specific function, and deviation from this function can cause disease. Thus, cell stability, or the preservation of cell identity, is fundamental for a functioning organism. Historically, it was believed that the cell genome changes with development ^(1)^. Genetic information unnecessary for the new cell identity and new function would be erased. John Gurdon challenged this consensus with his famous tadpole experiments, which demonstrated that the genome of a somatic cell contains all the necessary genetic information to create a fully functional living being ^(2)^. These experiments suggested that the genome is preserved and that epigenetics determine cell identity. They also suggested that cells can be reversed to an embryonic state. It took nearly another fifty years for scientists to reprogram cells to their embryonic state in vitro; we showed that the pluripotent network could be activated in mouse fibroblasts by exogenously expressing four transcription factors: *Oct3/4*, *Sox2*, *Klf4*, and *c-Myc* (OSKM) ^(3)^. Further, we showed that the same approach works in human fibroblasts ^(4)^. Following these initial reports, the number of species that have had their cells reprogrammed to the pluripotent state using OSKM has confirmed that the reprogramming mechanism is universal ^(5)^. The reprogrammed cells were named induced pluripotent stem cells (iPSCs) (Figure 1).

iPSCs are functionally equivalent to embryonic stem cells (ESCs) ^(6), (7), (8)^. Both represent the pluripotent stage of embryogenesis and have the capacity to differentiate into all germ layers, providing an in vitro model for scientists to study development. Pluripotent stem cells (PSCs), such as iPSCs and ESCs, have two attractive qualities for the study of organismal development and medical therapies. First is their indefinite capacity to proliferate, and second is their capacity to differentiate into all cell types of the body. Thus, they could theoretically provide unlimited numbers of any cell type desired. However, the creation of ESCs requires the destruction of embryos, which has stirred controversy and inconsistent policies that have discouraged ESC research ^(9)^. On the other hand, iPSCs can be acquired by reprogramming somatic cells, including those easily accessible and less controversial such as blood and skin cells. Although ESCs and iPSCs are viewed as functionally equivalent ^(10)^, because reprogramming intentionally disrupts a number of stable genomic and epigenomic networks, greater caution is advised when applying the latter to clinical use ^(11)^.

Nevertheless, the discovery of iPSCs has opened the door to a new generation of scientific research, including cell identity and organismal development, and medical research, including regenerative medicine, drug discovery, and disease modeling. In this short review, we focus on medical applications of iPSCs with discussion about efforts that have already led to clinical studies and those expected in the near future.

## Regenerative Medicine

It was only a year after the first iPSC report that scientists provided the first proof-of-principle for the therapeutic potential of these cells. The Jaenisch group used reprogrammed fibroblasts to treat a humanized sickle cell anemia mouse model ^(12)^. The autologous iPSCs were differentiated into hematopoietic progenitors and transplanted into the model, but beforehand had the disease-causing mutation corrected. Following transplantation, the mice were rescued of the disease. In 2008, one year after the first human iPSC study, researchers showed that patient cells could be reprogrammed into iPSCs by reprogramming the fibroblasts of an 82-year-old woman with familial amyotrophic lateral sclerosis (ALS) ^(13)^. These and other studies revealed that iPSCs could lead to a new generation of autologous cell therapies.

Indeed, in 2014, a Japanese team led by Masayo Takahashi announced the first autologous iPSC-based therapy for a 77-year-old female patient suffering from neovascular age-related macular degeneration (AMD). Skin fibroblasts were reprogrammed to iPSCs, which were differentiated into retinal pigment epithelial (RPE) sheets and transplanted back without the administration of immunosuppressants ^(14)^. AMD is a common degenerative retinal disease that leads to a loss of vision. Both eyes of the patient had AMD symptoms, but only the right eye received the transplantation; the left eye received injections of an anti-vascular endothelial growth factor (VEGF) drug, which is standard therapy. A 25-month follow-up revealed neither serious adverse events nor signs of rejection and that the degeneration had stopped in the right eye, confirming the safety and feasibility of iPSC-based autologous transplantation in humans. On the other hand, the left eye showed continued degeneration despite VEGF therapy.

As encouraging as the AMD study is, it also indicates two important caveats that must be resolved before iPSC-based therapies become universal and serve the treatment of multiple diseases, namely cost and time. The current cost of a single autologous iPSC-based therapy, which includes reprogramming to iPSCs, differentiating the iPSCs to the cells for transplantation, and the numerous quality checks, is prohibitive for wide patient care, whereas the time required could result in further degeneration of the patient’s state, making the treatment ineffective. In the above AMD case, the patient had to wait approximately six months between providing the fibroblast sample and receiving the transplant at a cost that approximated 1 million USD. To lower the cost and time, organizations have been manufacturing iPSC banks that are preparing clinical-grade allogeneic iPSCs. Several studies have shown that matching human leukocyte antigen (HLA) loci reduces the immune rejection and increases the survival rate of grafts of iPSC-derived neurons and cardiomyocytes in non-human primate models^(15), (16)^, validating the use of these cells pre-clinically. Depending on the targeted organs, immunosuppressants would still be needed because other HLA and non-HLA antigens stimulate natural killer T cells ^(17)^; however, as observed in the non-human primate models, the amount of immunosuppressant would be less.

**Figure 2.**
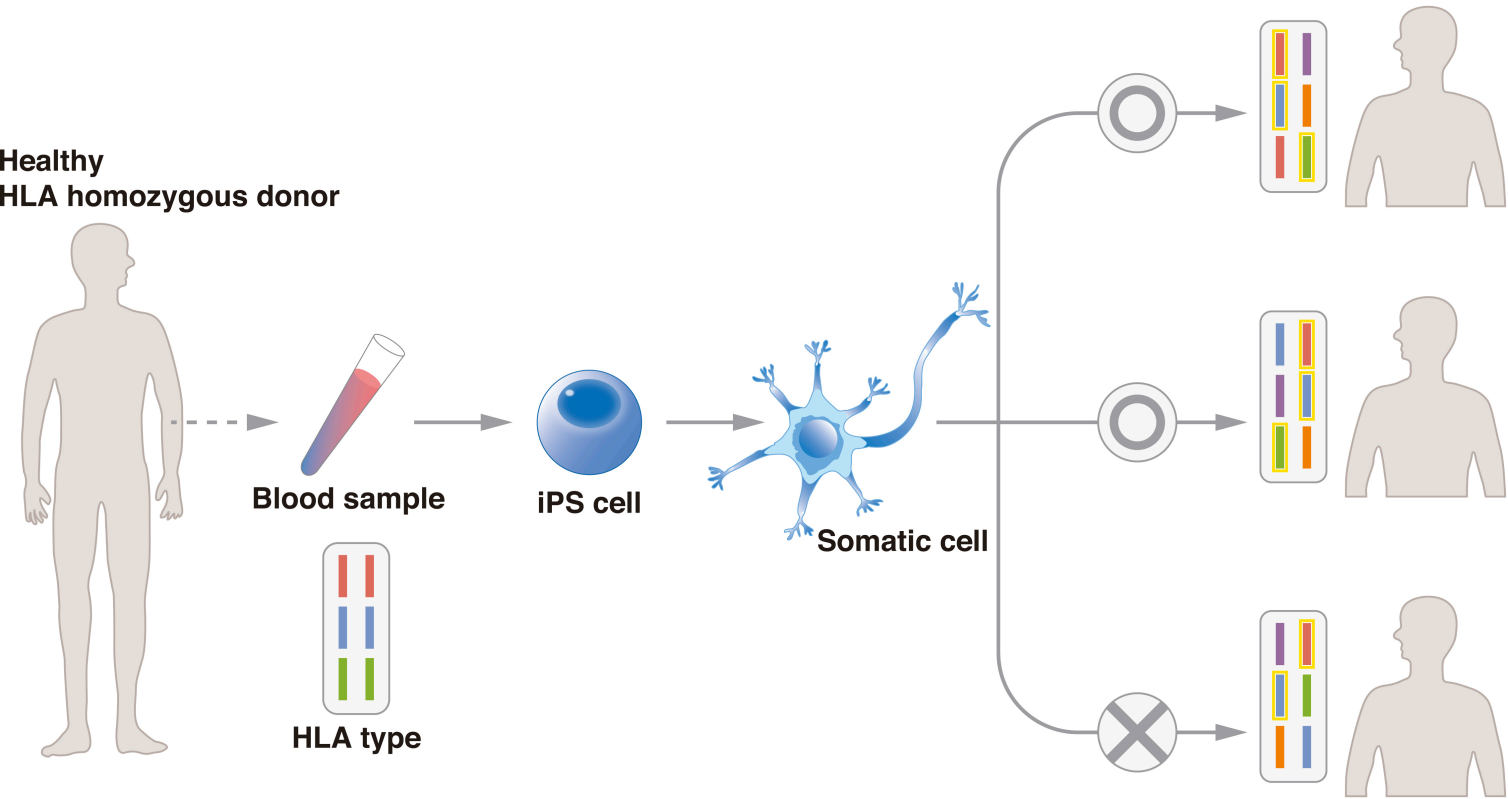
In iPSC banks such as the iPS Cell Stock for Regenerative Medicine, blood is taken from a super donor, i.e., a donor who is homozygous at the major HLA-A, HLA-B, and HLA-DRB1 loci. The cells are reprogrammed into clinical-grade iPSCs. The iPSCs are distributed to organizations that are conducting cell therapy. The use of super donor samples increases the probability of donor-recipient matching. In the figure, cells from the donor match two of the three patients, all of whom have different major HLA-A, HLA-B, and HLA-DRB1 haplotypes.

To promote the use of allogeneic iPSCs, the Center for iPS Cell Research and Application (CiRA), Kyoto University, started the iPS Cell Stock for Regenerative Medicine in 2013. In this project, CiRA generates clinical-grade iPSCs from samples of peripheral blood and umbilical cord blood from healthy “super donors.” The iPSCs are available to research institutes, pharmaceutical companies, and other organizations for regenerative medicine. Super donors are people with homozygous major HLA loci (Figure 2). To date, CiRA has distributed iPSCs with the three most common major HLA haplotypes in Japan, which would serve approximately 30% of the Japanese population, and aims to cover 50% by FY2020. Cellular Dynamics International, Inc., is establishing iPSC lines for cell therapy with a similar strategy, and it announced in 2015 that two cell lines had been generated that can match 19% of the U.S. population. Using cells from CiRA’s iPS Cell Stock, the researchers responsible for the first AMD study have begun a second therapy in which they have transplanted allogeneic iPSC-derived RPE sheets to five patients. Similar clinical studies are expected for several diseases, with announcements for clinical studies on Parkinson’s disease and cardiac failure having been made this year.

Another promising example of cell therapies using the iPS Cell Stock is platelet transfusion. Standardized platelet transfusions have been ongoing for more than half a century and have consistently depended on blood donors. However, despite the universality of this approach, it is anticipated that platelet demand will greatly outweigh platelet supply in several developed countries in the next decade. Scientists are therefore considering iPSCs as a way to prepare clinical-grade platelets at the industrial level to circumvent the dependency on blood donors ^(18)^. Because they are anucleate, platelets themselves can be stored only for a few days, which is why capricious donor availability can cause capricious platelet supply. Megakaryocytes are platelet uniprogenitors. These cells can be cryofrozen and cultured to release platelets, but they are extremely difficult to obtain from blood samples because of their negligible numbers ^(19)^. Taking advantage of iPSC technology, scientists have produced immortalized megakaryocytes at numbers feasible for clinical purposes ^(20), (21)^. One of the biggest challenges in platelet transfusions is the large quantity of platelets needed. The above AMD cell therapy required approximately 10^5^ cells, but the number for platelet transfusions is on the scale of 10^11^. The combination of iPSC-derived immortalized megakaryocytes and bioreactors, which can recapitulate the bone marrow environment in which megakaryocytes shed platelets, is producing platelets nearing this number^(22)^, with the expectation of clinical application in the next year or two.

Another realm where iPSC technology has exciting potential is in cancer immunotherapies. Adoptive T-cell therapy (ACT) involves harvesting T cells from a patient, culturing and expanding these cells, and finally infusing them back into the patient ^(23)^. T cells have many subtypes, including naïve, memory, and effector, and each serves a specific role in immunity and has its own capacity for proliferation and cytotoxicity. Upon chronic stimulation, which is the case in many cancers, T cells become exhausted, resulting in a condition in which T cells recognize the cancer antigen but are unable to exert any effective cytotoxic function ^(24)^. The expansion stage in ACT results in suboptimal T cells that also tend to show the exhausted phenotype ^(25)^. As a way to escape this phenotype, scientists have reprogrammed T cells to iPSCs. Expanding and differentiating these cells result in rejuvenated iPSC-derived T cells (T-iPSCs) that can take any of the above subtypes ^(26), (27)^. Unlike other iPSC-based therapies, in which the original reprogrammed cell type can be easily accessed like fibroblasts or blood, the original reprogrammed cell type in ACT must be T cells because otherwise random T-cell receptor (TCR) rearrangement may occur, thus losing affinity for the cancer antigen.

Cancer immunotherapies may benefit from iPSC technology in another way. Chimeric antigen receptors (CAR) were first proposed 25 years ago ^(28)^. This concept combines the extracellular domains of an antibody with the intracellular machinery of T cells, which results in cells that have receptors with high affinity for cancer cells and the immune activity of T cells. Currently, CAR T-cell therapies are best suited for blood cancers, and in 2017, the FDA approved two CAR T-cell therapies, one for acute lymphoblastic leukemia and the other for advanced lymphomas ^(29)^. The promise of CAR therapies excludes concerns about TCR rearrangement; thus, T-iPSCs are not necessary. However, iPSC technology could advance CAR T-cell therapies in other ways. Evidence has shown that the effectiveness of CAR T-cell therapies correlates with the inclusion of naïve and memory T cells ^(30), (31)^. Through iPSC technology, it is theoretically possible to prepare the appropriate subtypes for optimal CAR T-cell therapy. Current CAR T-cell therapies cost nearly 500,000 USD ^(32)^, which, like the autologous AMD iPSC therapy, makes it difficult to provide through national health insurances. Encouragingly, one preliminary study has confirmed that the two technologies are compatible ^(33)^, suggesting that iPSC banks, like CiRA’s iPS Cell Stock, could contribute to lowering the cost.

In contrast to the anticipation of iPSC clinical therapies for AMD and other diseases, many more clinical studies that use ESCs as the cell source are currently planned ^(34)^. ESCs and iPSCs have their own advantages and disadvantages: ESCs do not undergo extensive epigenetic reprogramming, whereas iPSCs can be generated from adults whose medical history and HLA haplotype information are available.

## Drug Discovery

**Figure 3.**
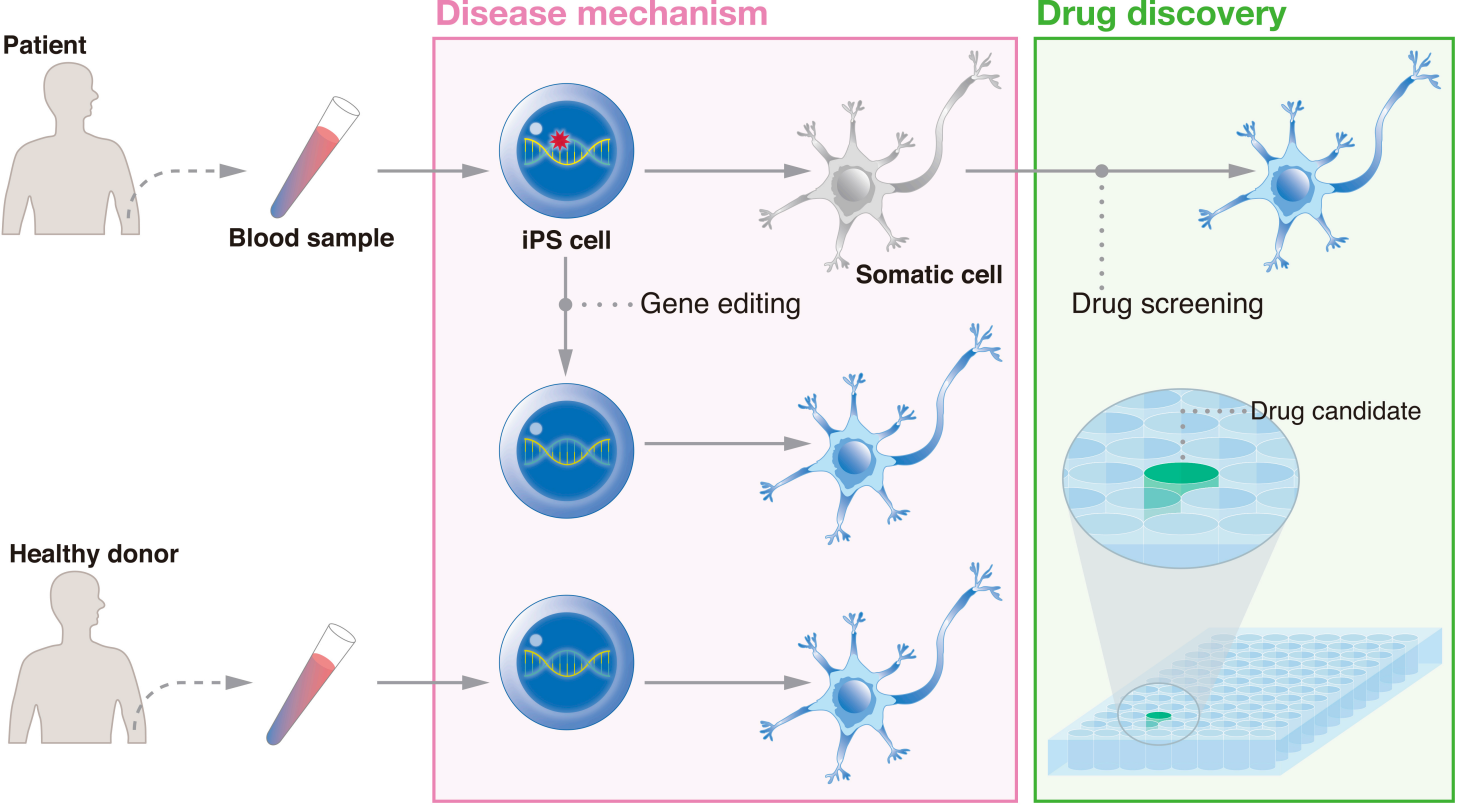
iPSCs provide a unique human model for drug discovery. Because patient cells can be reprogrammed to iPSCs and differentiated toward the diseased cell type, researchers can compare the differentiation process between patient cells and healthy donor cells and/or gene-corrected patient-derived cells to identify novel targets. Drug screening can be conducted to find candidates that alleviate the disease phenotype.

In addition to regenerative medicine, iPSCs have unique features that make them attractive for another medical application, i.e., drug discovery (Figure 3). The benefits of drug discovery come in part from the advantages of using iPSCs to model the disease development process. Many diseases are both idiopathic and intractable. For some diseases, the majority of cases are sporadic; ＞95% of Alzheimer’s disease patients are spoardic ^(35)^. In addition, symptoms may mark a progression of the disease to a point where only extremely invasive measures can lead to recovery. For example, in Parkinson’s disease, approximately half of the patients’ dopaminergic neurons are already lost at the time of diagnosis ^(36)^. By reprogramming patient cells to the pluripotent state and then differentiating iPSCs to the affected cells, researchers can detect irregularities that contribute to the disease even if the genetic cause is unknown.

One of the first studies that used patient iPSCs to model a disease reprogrammed fibroblasts from two females and one male suffering from familial dysautonomia ^(37)^. The iPSCs were differentiated to the neural crest lineage. Those from patients showed deficiencies in the expression of genes related to peripheral neurogenesis and neuronal differentiation, consistent with other models of the disease. Importantly, the investigators showed the effects of the plant hormone kinetin on the iPSC-derived cells, providing the first study that tested chemical compounds on patient iPSC products. Research on neurodegenerative diseases using patient iPSCs have already led to several dozen drug candidates ^(38)^.

Some of these studies have even progressed to the clinical stage. The Eggan lab prepared iPSCs from ALS patients with mutations in *SOD1*, which is found in 20% of familial ALS cases and 1%-4% of all ALS cases ^(39)^. Motor neurons differentiated from patient iPSCs showed dysfunctional potassium channels that could be corrected with ezogabine, a Kv7.2/3 potassium channel agonist that has been approved for epilepsy treatment. This discovery is a prototype of the medical advances that could come from iPSC research. First, the observation that the Kv7.2/3 potassium channel can act as a target to treat the disease had never been realized previously ^(40)^. Second, the cost and time of drug discovery is pushing industry to consider alternative strategies. Drug repositioning, where approved drugs are tested for unapproved treatment, as exemplified by ezogabine, is estimated to reduce both the cost and time of reaching market to one-third ^(41)^.

Another example of drug repositioning through iPSC-based disease modeling comes with study of the skeletal system. Fibrodysplasia ossificans progressiva (FOP) is an extremely rare genetic disorder, with only several hundred cases reported worldwide. In this disease, ectopic bones are formed in muscle and connective tissues. Mutations in *ACVR1* cause abnormal BMP signaling ^(42)^, but the precise mechanism is not well understood. Interestingly, a study using patient iPSCs found that the excessive BMP signaling is induced by Activin-A, a ligand associated more with TGF-β signaling than BMP signaling ^(43)^. A subsequent study by the same group identified rapamycin, an mTOR inhibitor approved as an immunosuppressant, as a candidate drug for the disease ^(44)^. A clinical trial for rapamycin as treatment for FOP based on these findings began in Japan in 2017.

Insights using iPSCs from patients with skeletal dysplasia have also led to potential drug repositioning. Here researchers prepared iPSCs from patients suffering from achondroplasia, a monogenic form of dwarfism ^(45)^. As expected, chondrogenic differentiation from patient iPSCs was impaired. Intriguingly, the addition of statin, a well-known cholesterol-lowering drug, to the differentiation protocol rescued cartilage differentiation. Using this information, the same study tested statin in a mouse model of achondroplasia, finding that administration of the drug led to normal bone growth without affecting bone growth in wild-type mice.

Because they are already approved drugs, there is ample patient data on ezogabine, rapamycin, and especially statin, which will lower the costs of pre-clinical studies, as safe doses and side effects have been extensively examined. These advantages have promoted studies that investigate the synergistic effects of multiple drugs, as recently reported for Alzheimer’s disease ^(46)^. The study found that the combination of bromocriptine, topiramate, and cromolyn, which are respectively approved to treat Parkinson’s disease, epilepsy, and asthma, maximally reduced the production of amyloid β peptide in vitro, whose aggregates form the hallmark amyloid plaques of Alzheimer’s disease. Excitingly, this effect held true for cortical neurons derived from the iPSCs of patients with different causative mutations and even sporadic cases.

Besides drugs, in recent years, investigators have considered the possibility of using antibodies to counteract the plaques found in Alzheimer’s disease, as antibodies for amyloid β and extracellular tau have showed promising results in stopping the spread of plaque formation in vitro, in animals and in patients ^(47), (48)^. There is controversy in the causal relationship between amyloid β and extracellular tau. One study that prepared iPSCs from four familial and one sporadic Alzheimer’s disease patients found positive feedback between the two substances and that an antibody for extracellular tau but not full-length tau could reduce amyloid β levels in vitro and in mouse models ^(49)^. These studies have led to a clinical trial for antibody treatment against the disease.

Classical drug discovery relies on target-based screening, but this strategy is difficult when the molecular cause is unknown. Thus, there has been a shift to phenotypic screening, which has a higher probability of identifying compounds for unknown targets or molecular mechanisms ^(50)^, with one example being the use of rapamycin to treat FOP, as described above. iPSCs are appropriately suited for this shift and have therefore captured the attention of industry. Most academic-industry collaborations involve the industry sending its researchers to the academic institutions. In contrast, one industry in Japan has built infrastructure at its own site where academic and industrial researchers together conduct their iPSC experiments ^(51)^. The hope is that this partnership will become a new model for future collaborations.

Phenotypic screening is expected to have a great impact on therapies for complex diseases such as autism spectral disorder (ASD). ASD has been associated with mutations, mostly rare, in hundreds of genes ^(52)^. iPSCs from patients have been used to explore genes commonly mutated in ASD, such as *CHD8*, and to discover novel genes associated with ASD, such as *TRPC6* ^(53), (54)^. However, even though the differentiation of iPSCs to specific neural cell types have led to the discovery of drug targets, researchers recognize that because the differentiation cultures do not allow cells to develop in their natural microenvironment, the observed cell phenotypes may not be reflective of the patient condition ^(55)^. This concern is especially true when the disease pathogenesis is the result of degenerated cell networks rather than degenerated cells.

To capture the phenotypes of these diseases more accurately, researchers are investigating 3D organoids. These structures are aggregates that self-organize from the culturing of a single or multiple progenitors and are expected to better recapitulate cell networks than assemblies of different cell types. Already, 3D organoids have been prepared from iPSCs to study diseases in several different organ systems ^(56)^. In combination with iPSC technologies, organoid research has helped to better understand how Zika virus disrupts neurodevelopment and led to multiple candidate compounds that could ameliorate the brain damage caused by the virus ^(57)^. Using iPSCs from patients with severe idiopathic ASD, the Vaccarino group found that telencephalic organoids, whose formation mimicked first-trimester development, had excessive numbers of GABAergic inhibitory neurons due to the overexpression of *FOXG1* ^(58)^. This novel finding was common among patient cells from four families that were genotypically heterogeneous but phenotypically similar.

Finally, while it has not led to the discovery of any new drugs yet, iPSC technology provides an attractive system for the study of cancer ^(59)^. Many cancers are the result of environmental factors such as a viral or bacterial infection, which induces aberrant epigenetics with few changes to the genome. Likewise, cell reprogramming induces epigenetic change without genetic change. Studies have shown that in vivo reprogramming of cancer cells can result in non-neoplastic cells dependent on the organ type, reaffirming the influence of the environment ^(60), (61)^. These studies can be of special advantage for pediatric cancers, which show far fewer genetic mutations than would be expected from their diverse clinical presentation ^(62)^. Further, the ability to reprogram cancer cells in vivo will allow the formation of new models that will help identify how epigenetics and genetics cooperatively determine whether a cell will become cancerous or remain in a benign state. Such research could contribute to new epigenetic targets for anti-cancer drugs.

## Conclusion

It is quite remarkable that in just over 10 years, research using iPSCs has led to several clinical studies, with many more expected to follow. The mass production of clinical-grade iPSCs through projects such as the iPS Cell Stock for Regenerative Medicine is expected to make iPSC therapies available to a large population at affordable costs. Moreover, the ability to generate iPSCs from patient samples has resulted in new in vitro human disease models, providing novel insights on early molecular events that regulate the pathogenesis.

In response to these features, there is much anticipation about where iPSC research will lead, but the endpoint will depend on more than science. While iPSCs circumvent several of the ethical controversies burdening ESC research, they also bring their own unique set of bioethics that was once only conceived by science fiction. Great progress has been made in inducing the differentiation of mouse PSCs to sperms and oocytes, which should act as a basis for doing the same with human PSCs ^(63)^. The possibility of producing sperms and oocytes from one’s blood through iPSC technology would provide new models and therapies for infertility. They also introduce the possibility of same sex couples no longer needing a third party because both sperm and egg could be made from the partners regardless of gender. Another potential use of PSCs is to create organs for transplantation. In theory, by transplanting human PSCs into early embryos of large animals, one can generate an organ composed mainly of human cells inside the animal ^(64)^. Indeed, such xeno organs have been reported in other species ^(65)^. These emerging technologies should be openly discussed from ethical, legal, and social points of views.

There are still a number of challenges that must be overcome for iPSCs to reach their full potential. For example, more efficient reprogramming methods and automated culture systems could make autologous therapies practical, and methods that minimize clonal variations of iPSCs would provide higher quality cells for clinical therapies. Quality control is another challenge, and we have been routinely using whole-genome sequencing to evaluate each iPSC line, but predicting cancer risk based on sequence information is a formidable task. Even with high quality and safe iPSCs, advances in differentiation protocols, not only for each cell lineage but also for more complex 3D structures, tissues, and organs are needed. These challenges show that iPSC research will benefit from scientists of various fields working together to realize innovative iPSC-based treatments and medicines.

## Article Information

### Conflicts of Interest

Shinya Yamanaka holds a scientific advisory role in iPS Academia Japan, Inc., without salary.

### Acknowledgement

The authors thank Masaya Todani for the figure illustrations and other CiRA colleagues for their helpful discussions.

### Author Contributions

Peter Karagiannis and Ayaka Nakauchi contributed equally to this work.
