# Erratum for DOI: 10.31662/jmaj.2018-0005

**DOI:** 10.31662/jmaj.e0001

**Published:** 2018-10-31

**Authors:** Peter Karagiannis, Ayaka Nakauchi, Shinya Yamanaka

**Affiliations:** 1 Center for iPS Cell Research and Application, Kyoto University, Kyoto, Japan

Article: Karagiannis P, Nakauchi A, Yamanaka S. Bringing Induced Pluripotent Stem Cell Technology to the Bedside. JMA J. 2018;1(1):6-14.

Correction: Reference 15.

Original: Kikuchi T, Morizane A, Doi D, et al. Human iPS cell-derived dopaminergic neurons function in a primate Parkinson’s disease model. Nature. 2017;548(7669):592-6.

Corrected: Morizane A, Kikuchi T, Doi D, et al. MHC matching improves engraftment of iPSC-derived neurons in non-human primates. Nat Commun. 2017;8(1):385.

